# Social and economic impact of diabetics in Bangladesh: protocol for a case–control study

**DOI:** 10.1186/1471-2458-13-1217

**Published:** 2013-12-21

**Authors:** Sheikh Mohammed Shariful Islam, Andreas Lechner, Uta Ferrari, Guenter Froeschl, Louis W Niessen, Jochen Seissler, Dewan Shamsul Alam

**Affiliations:** 1 Center for Control of Chronic Diseases (CCCD), icddr,b, and Center for International Health, Ludwig-Maximilians-Universität, Munich, Germany; 2 Diabetes Center, Diabetes Research Group, Medizinische Klinik und Poliklinik IV, Ludwig-Maximilians-Universität, Ziemssenstr. 1, München 80336, Germany; 3 Diabetes Center, Diabetes Research Group, Medizinische Klinik und Poliklinik IV, Ludwig-Maximilians-Universität, Ziemssenstr. 1, München 80336, Germany; 4 Center for International Health, Ludwig-Maximilians-Universität, Munich, Germany; 5 Liverpool School of Tropical Medicine, UK and Adjunct Professor, Johns Hopkins University, Baltimore, MD, USA; 6 Diabetes Center, Diabetes Research Group, Medizinische Klinik und Poliklinik IV, Ludwig-Maximilians-Universität, Ziemssenstr. 1, München 80336, Germany; 7 CCCD, icddr,b, 68, Shaheed Tajuddin Ahmed Sarani, Mohakhali, Dhaka 1212, Bangladesh

**Keywords:** Diabetes, Social and economic impact, Bangladesh

## Abstract

**Background:**

Diabetes affects both individuals and their families and has an impact on economic and social development of a country. Information on the availability, cost, and quality of medical care for diabetes is mostly not available for many low- and middle-income countries including Bangladesh. Complications from diabetes, which can be devastating, could largely be prevented by wider use of several inexpensive generic medicines, simple tests and monitoring and can be a cost saving intervention. This study will provide an in-depth and comprehensive picture of social and economic impacts of diabetes in Bangladesh and propose clear recommendations for improving prevention and management of diabetes. The objectives of the study are:

1) To study the association between diabetes and other health problems and its social impacts

2) To estimate the economic impact of diabetes including total direct and indirect costs

3) To measure the impact of diabetes on quality of life among diabetes patients in Bangladesh

4) To study the impact of diabetes on the health care system

**Methods:**

This is a case–control study comparing cases with type 2 diabetes to controls without diabetes matched on age, sex and place of residence. 564 cases and 564 controls will be selected from the outpatient department of a tertiary hospital in Dhaka, Bangladesh. Data on socioeconomic status, health utility index, direct and indirect costs for diabetes, medication adherence, quality of life, treatment satisfaction, diet, physical activity, mental state examination, weight, height, hip and waist circumference, blood pressure, pulse, medication history, laboratory data and physical examination will be conducted.

Outcome measures: The primary outcome measures will be association between diabetes and other health problems, cost of diabetes, impact of diabetes on quality of life and secondary outcome measures are impact of diabetes on healthcare systems in Bangladesh.

**Discussion:**

This study will provide an in-depth and comprehensive picture of social and economic impacts of diabetics in Bangladesh and propose clear recommendations for improving prevention and management of diabetics. It will help to develop programs and policies for better management of Diabetics and cost effective strategies in Bangladesh context.

## Background

Diabetes can lead to a variety of disabling, life-threatening, and expensive complications, including stroke, heart attack, renal disease, neuropathy, peripheral artery disease, lower-limb amputation, and visual impairment. In 2011, diabetes was associated with 4.6 million deaths worldwide, causing more deaths than HIV and malaria combined and consumed at least 465 billion current U.S. dollars in health care resources [1,2]. Demographic transition, combined with urbanization and industrialization, has resulted in drastic changes in lifestyles globally. Consequently, lifestyle-related diseases like diabetes have emerged as major public health problems in the developing countries [3-5]. Three-quarters of persons with diabetes live in low- and middle-income countries [2] where more than 80% of diabetes related deaths occur. It is estimated that by 2030, Bangladesh will be the 5th largest country in the world in terms of population living with diabetes which will be challenging its health system in terms of meeting the demands of its population [2].

In Bangladesh, diabetes affects all segments of the population and is one of the leading causes of premature morbidity and mortality, and requires life-long healthcare services [6]. Poverty, low education, and gender discrimination adversely affect women with diabetes [7]. The levels of knowledge about diabetes among the at-risk population and among those who suffer from the disease are poor [3]. About 90% of all respondents did not test their blood glucose regularly and suffered from several complications with low medication adherence and poor clinic attendance [3]. Information on the availability, cost, and quality of medical care for diabetes is generally not available in Bangladesh. Complications from diabetes, which can be devastating, could largely be prevented by wider use of several inexpensive generic medicines, simple tests and monitoring and can be costs saving interventions in a country like Bangladesh [8].

Diabetes imposes a considerable burden on health systems and societies [9]. The cost implications of diabetes to society are multi-fold: direct costs to people with diabetes, their families and to the health care sectors, indirect costs to society and government, which are the productivity costs; and intangible costs, which means adverse effects on quality of life [10]. Very little is known about the economic impact of diabetes in the developing world where predicted increases in prevalence are highest [11]. With increasing concern among providers about the rising costs of health care, economic assessments of the impact of various diseases are growing in importance [12]. In assessing the economics of diabetes, however, a number of questions must be addressed, including the definition and measurement of incidence, the definition and measurement of economic impact, and a method by which incidence and economic impact may be linked [13]. Diabetes clearly affects individuals, families, providers of medical care, and the governmental ministries that pay for care. These effects probably also disable communities and retard national economic growth. However, until basic data have been gathered, the magnitude of these effects can barely be estimated.

The economic and human costs provoked by diabetes in a large population such as in Bangladesh will be substantial. However, despite these astounding numbers of diabetes population in Bangladesh which is expected to rise to more than 16 million by 2020 [2], there are little evidence based research on the social and economic impact of diabetics in Bangladesh. In this context, we propose to conduct a first-ever comprehensive case–control study of social and economic impact of diabetes in Bangladesh.

### Hypothesis

1. The total annual per-person expenditures for medical care among persons with diabetes would exceed total annual per-person expenditures among persons without diabetes.

2. The quality of life for people without diabetes would be better than people with diabetes.

### Objectives

The main objective of this study is to describe the social and economic impact of diabetes in Bangladesh, including out-pocket expenditures by patients and their families; other expenditures for medical care; impact on quality of life, disability and health utility; impact on the income, employment, education, and property of patients and their families; and the efficiency of medical care received for diabetes. The specific objectives are as follows:

1) To study the association between diabetes and other health problems and its social impacts

2) To estimate the economic impact of diabetes including total direct and indirect costs

3) To measure the impact of diabetes on quality of life among diabetes patients in Bangladesh

4) To study the impact of diabetes on the health care system

## Methods

This is a case–control study comparing cases with type 2 diabetes to controls without diabetes matched on age and sex. The case–control study design isolates the costs caused by diabetes by revealing differences and ratios between persons with diabetes and matched controls. The method solves the otherwise very difficult problem of allocating joint costs-for example costs for a heart attack admission that are partly caused by diabetes but also partly due to other causes.

Data will be obtained by personal interviews with the cases and controls. Interview data will be supplemented with data from health facilities to more fully describe the subjects’ costs of care. Searching of health facility records will be based on personal-interview responses that indicate when and where subjects have received medical care. The form for unit costs data collection from health facilities will be piloted in a similar setting in Bangladesh before the study. All subjects will provide informed consent prior to being interviewed and having their health facility records examined.

### Study population

This study will be carried out among patients attending the outpatient department of the Bangladesh Institute of Research & Rehabilitation in Diabetes, Endocrine and Metabolic Disorders (BIRDEM) Hospital. BIRDEM has the largest diabetic Out-Patient Department (OPD) turnover in the world under a single roof and it has an In-Patient hospital with about 700 beds with all modern disciplines of medicine [14]. BIRDEM is the only national level tertiary health care and research institute for diabetes in Bangladesh and recognized as WHO collaborating center for research on prevention and control of diabetes. The OPD of BIRDEM currently has 497756 registered diabetes patients and serves more than 4000 patients daily [15]. Patients come here for treatment from all over the country. The average number of patients per day in the BIRDEM OPD will be sufficient to cover the total sample size of this study within the given time frame.

### Sample size and statistical power

Calculations of the economic impacts of diabetes on individuals cannot adequately describe the burden or impact on social and national economics of diabetes, for three reasons. First, their assessments of the economic burden of diabetes are confounded by competing risks and co-morbidities. Because there are common risk factors that increase one’s risk of multiple diseases (e.g. tobacco use or obesity that could be linked to diabetes, heart disease or cancer), unadjusted estimates of the economic burden attributed to any one condition are likely to be upwardly biased. Second, socioeconomic and demographic differences across households can influence healthcare expenditures and other coping strategies in response to illness. Finally, aggregate calculations also include impacts of diabetes on households unaffected by diabetes, such as via an overall slowing of economic growth that lowers employment rates and incomes [16]. Considering all these options, we focused specifically on selecting cases with diabetes and age group, sex and place of residence matched controls. We used the following formula to calculate the number of cases and controls [17]:

$$n=\left(\frac{r+1}{r}\right)\frac{\sigma^{2}{\left(Z_{\beta }+Z_{\alpha 2}\right)}^{2}}{{\left(d\mathrm{ifference}\right)}^{2}}$$

– For 80% power, Z_β_=.84

– For 0.05 significance level, Z_α_=1.96

– r = 1 (equal number of cases and controls)

– σ=12.0 (Standard Deviation of the outcome variable)

– Difference = 2.0 (Effect size of the mean difference)

$$n=\left(2\right)\frac{12^{2}\left(7.84\right)}{{\left(2\right)}^{2}}=\left(2\right)6^{2}\left(7.84\right)=564$$

### n = 564 (564 cases and 564 controls)

Therefore, we will recruit a total 1128 participants (564 cases and 564 controls) for this study which should be sufficient to conduct different sub-group analysis to measure the economic burden of diabetes across different socio-economic groups.

### Primary outcome measures

•Association between diabetes and other health problems (Questionnaires)

•Cost of diabetes (Questionnaire and statistical modelling)

•Impact of diabetes on quality of life (Questionnaires and statistical modelling)

### Secondary outcome measures

•Impact of diabetes on health care systems (questionnaires and statistical modelling)

### Inclusion and exclusion criteria

#### Eligibility

Ages: 20–60 years, Genders: All genders.

#### Inclusion criteria for cases

•Diagnosed as type 2 diabetic patient at BIRDEM OPD according to WHO criteria

•Willing to answer the questionnaire and provide anthropometric measurements

•Willing to have health records checked by the study team

•Providing written informed consent.

#### Exclusion criteria for cases

•Cases younger than 20 years or older than 60 years

•Patients with serious co-morbid conditions requiring immediate hospitalization or having mental illness

•Patients unable to give their informed consent

#### Inclusion criteria for matched controls

•Individuals with similar sex and age range (within 5 years band) with cases

•No history of diabetes

#### Exclusion criteria for controls

•A known previous history of any diabetes (self-reported or physician diagnosed)

•Unable to provide consent.

### Sampling design

#### Case selection and recruitment

Cases with diagnosed diabetes will be selected from the Bangladesh Institute of Research on Diabetics, Endocrinology and Metabolism (BIRDEM) Hospital outpatient department (OPD) where patients from all over Bangladesh comes for management of diabetes and its complications. We will identify cases with diabetes from consecutive patients as they come for treatment. Potential subjects will be asked if they are willing to participate in the study and, if so, a time and location for a personal interview will be arranged and contact information gathered.

#### Selection and recruitment of control subjects

Controls will be individuals ‘frequency-matched’ to cases on age (within 5 years band) and sex (male or female), without a self-reported history of diabetes (or diagnosed as diabetes by a physician). They will be recruited within 48 hours of recruiting the index case, from either (i) the same geographical residence of cases, or (ii) visitors of patients attending the out-patient department, or (iii) non-blood related visitors of index diabetes cases, in the same hospitals,. All controls will undergo identical study questioning and examination as cases. One completed control interview will be obtained for each case interview.

While we are recruiting case subjects and scheduling personal interviews, we will ask cases who have said they want to participate to identify five persons living closest to him or her who are of the same age (+/− 5 years) and sex. For each of these potential control subjects, we will ask the case to provide whatever contact information that he or she can. Cases will be assured that their health information will not be shared with the control. We will then randomly order the names on the resulting list of five and approach each of these individual’s households in order, by telephone if possible or, if not, by visiting their households. If the first-ordered individual is at home, the interviewer will recruit this individual on the spot and obtain informed consent and complete the interview immediately if this is practicable. Otherwise, if the individual is not willing to be interviewed at the moment, an appointment for a later time will be made. If the first-ordered individual is at home but does not wish to participate, the interviewer will proceed to the household of the second-ordered individual and repeat the process.

If the second-ordered individual is not at home, the interviewer will attempt to get telephone contact information for this person so that recruitment can occur by phone at a later time, and an interview scheduled. Interviewers should not leave the area where they recruited the case without a lead to a potential control. If by the second household the interviewer has not recruited and interviewed a control, she/he will proceed to each of the remaining households to obtain contact information and gauge potential interest.

The approach described above is chosen because it can potentially achieve a balance between feasibility and scientific rigor and should be scalable in Bangladesh. By contrast, whereas use of population-based community controls may be desirable in principle, it is considerably more labour-intensive and expensive and cannot guarantee that such controls will necessarily represent the catchment areas from which hospital-based cases are derived, particularly since referral patterns to hospitals are complex. Furthermore, as controls in this study will be drawn from visitor of patients attending the out-patient department and non-blood related visitors of diabetes cases, it is expected that such approach would minimize, at least in part, major limitations involved with conventional hospital-based controls if we cannot recruit community based controls.

#### Use of surrogates

When a case or control subject is not able to speak or recall sufficiently completing the interview on their own, the participation of surrogate respondents will be allowed. The interviewer will ask another family member to answers the survey questions on behalf of the respondent. The name of the family member who answered the questions will be written in the survey and the surrogate code entered on the survey.

#### Non-response, non-completion, termination

If it proves impossible to complete an interview once it has begun, and it cannot be completed on another day, this case or control will be removed from the study sample and replaced with another person per protocol. They will also be labeled a “non-completer.” If a subject desires to terminate an interview prior to completion, the same procedures will be followed as for a non-completer. All data except for recruitment information obtained prior to commencing the interview will be destroyed. The interviewer will terminate the interviews of all control subjects who say that they have been diagnosed with diabetes, or whenever in the interviewer’s judgment the subject is not responding truthfully or accurately or no longer wishes to participate in the study.

#### Data collection tools

The research tools and instruments will include pre-defined questionnaires, anthropometric measurements, laboratory and medication lists, and physical examination. We will use the research tools developed by the International Diabetes Federation (IDF) Health Economic Group and translate them into Bengali as per WHO standards. All interviews will be conducted by trained interviewers under the supervision of a Research Physicians.

Hospital data gathering tools: Information on cost incurred by the hospitals is a vital component of illness costs. Information on treatments received by patients during their hospital visits is also critical in understanding utilization of health care. To generate cost incurred by third parties and to gather hospital information, we have developed a hospital unit costs tool to gather information on average costs including those incurred by third parties (by type and reference level of the hospital or outpatient clinic).

Estimation of expenditures for medical care will be based on participant recall to ascertain charges for medicines, supplies, and medical care services. To increase accuracy of recall, the interview schedule will only ask about events occurring only during the previous 90 days and attempt to improve temporal accuracy by asking respondents to name and associate a well-remembered event that had occurred approximately 90 days previously. To estimate expenditures for medicines, interviewers will ask subjects about their most recent purchase of each of the medicines they were currently using. For overnight admissions to hospital and OPDs, respondents will be asked to recall their total point-of-service payment, including charges for medicines and tests received or, if they paid only a portion out of pocket, the total bill or charge. Total charges per visit or hospital admission usually includes charges for medicines because doctors and hospitals typically use pharmaceutical sales as a primary means to finance operations. This subset will be used to estimate the characteristics of all events of the same kind, including mean length of hospital stay and mean payments per admission, per OPD visits, and per purchase of medicine.

#### Field testing

The research instruments and questionnaires and will be translated in Bengali and field tested in a similar setting to our current study site and population like the outpatient department of Bangladesh University of Health Science (BUHS) Hospital, Mirpur, Dhaka before conducting the research. 25 cases and 25 control subjects will be enrolled in the field testing. Feedback from the field testing will be used to improve the language and contents of the questionnaire and tools.

#### General outline plan for field work/data collection

On a typical day, the field staff will be present at the study site early in the morning when patient registration starts in the BIRDEM OPD. Registered patients who agree to participate in the study will be referred by the physician on duty to the study team. The study team will explain about the study in details to the participants and request for signing the informed consent to all who agree to participate in the study on a volunteer basis. The study team will also collect data from one control against one case.

On an average, 5–6 patient forms should be completed at the study site every day and the total data collection should be completed by 12 months. At the end of the day all team members will ensure that the completed data collection forms and instruments are properly stored in the field site. Data will be entered in an excel sheet in an ongoing basis. A log book of all participants with codes will be maintained by the study team. Written informed consent will be obtained from all participants that they agree to participate in the study voluntarily and are free to stop participating at any point of the study.

#### Follow-up plans

Quality Monitoring and Assurance: Data collection forms will be checked on a daily basis to ensure complete and accurate data entry by field research workers. Cross checking of data collection forms will be performed in the fields under supervision of the Research Officer for inconsistency and missing data. The Principal Investigator (PI) will be responsible for carrying out periodic data checks and he will look for systematic patterns, errors, scheduling problems or incomplete forms to provide feedback to the study nurse and to ensure integrity of data captured. Every week when the team meets, the PI will pick 2 questionnaires filled in the current week by one interviewer. The PI will go through the questionnaire, question by question with the entire team, to identify incomplete/missing entries and any errors. The purpose of this is to rectify and clear any doubts which the interviewers may still have.

The PI will visit the fields at least once every week and observe complete data collection by the field research assistants. He will provide his feedback and provide on-the-job training as necessary. Co-PIs and Co-Investigators will also visit the field site without prior notice to monitor data collection, physical measurements and biological sample collection, transportation, storage and analysis in accordance with the protocol. The instruments for physical measurements will be regularly calibrated according to guidelines of its manufacturers in the fields and a written record maintained which will be periodically checked by the PI for consistency and validity of data.

We will attempt to replicate interview and physical measurements in a random sub-set (~5%) of the sample to evaluate repeatability. For repeatability studies, we will have about 50 participants, 25 cases and 25 controls, with duplicate measures which will be sufficient to estimate kappa coefficients of agreement of inter and intra-observer reliability with a high degree of precision even for categorical variables. The participants will be selected among study participants who come to BIRDEM OPD for treatment and follow up and agree for the process.

#### Description of variables

•Socio-demographic and economic information: We will collect data on participant’s age, sex, religion, education, marital status, occupation, income, family size, family income, objects owned

•Knowledge of prevention, management and complications of DM: Levels of knowledge and perceptions of diabetics, Risk Factors for diabetics, knowledge about prevention, management and complications of diabetics.

•Self-perceived Quality of Life (OoL): This will be measured using EQ-5D 3 L, which is a standardised instrument for use as a measure for health outcome. It is widely used, and by many governments a recommended instrument for measuring health related QoL [18].

•Self-reported diseases: This will be based on self -reported information on diabetes, cardiovascular diseases, renal disease, stroke, arthritis, chronic respiratory disease, mental and physical disability by the study participants.

•Medical History: Any known history of diabetes and its type, CVD, hypertension, cancer, hypercholesterolemia, stroke, kidney diseases, other vascular disease, infectious disease, major surgery.

•Tobacco Use (WHO modified): A modified WHO questionnaire on use of different forms of tobacco currently and in the past will be recorded. Expenditure on tobacco use will also be recorded [19].

•Physical Activity (GPAQ): WHO GPAQ questionnaire which has been validated in the fields will be used to capture information on different forms of physical activity in MET which will be analyzed as per data analysis protocol [20,21].

•Diet: A modified WHO STEPS questionnaire will be administrated to ascertain food consumption pattern and dietary choices, type and frequency of consumption of different foods.

•Health seeking behavior: Data will be collected on type of health care practitioner visited during the past year, number of visits, distance travelled for consultation and expenditure for health.

•Use of medical services: Hospital and non hospital visits for health problems, medical tests performed.

•Impact of health problems: affecting personal and family life.

•Diabetes history: description of diagnosis, duration, self-management of diabetes.

•Mental health status (Patient Health Questionnaire, PHQ9): The PHQ 9 which is a standard toll for collecting mental health status in primary health care setting will be used in this study[22,23].

•Life events and Disability: Life events related questions like new job, marriage, separation, injury, accident in the family will be asked, which will be based on validated questionnaire.

•Anthropometric Measurements: Weight, Height, Waist and Hip circumference, Blood Pressure (BP), Pulse rate will be measured.

•Laboratory data: data on HbA1c, FBS, 2HAFB, S. Creatinine, Lipid Profile, ECG will be collected from patient record book as available.

•Physical examination: General examination of eyes, foot and neurology tests performed by a specialist will be recorded.

### Details about physical measurement

#### Measurement of blood pressure and pulse rate

Blood pressure is taken to determine the prevalence of raised blood pressure in the population. We will use Omron SEM-1 (Omron, Matsusaka Co., Japan) digital blood pressure measurement instrument which is widely used for research purpose in the community. Both systolic and diastolic blood pressure will be measured for each participant. Three repeated measurements will be recorded after an interval of 5 minutes alternating right and left hand.

#### Height

Height will be measured for all participants using portable stadiometer. Standing height is measured with the subject in bare feet, Back Square against the wall and eyes looking straight ahead. A set square resting on the scalp and a tape measurement from the wall/bed is used to measure height to the nearest 0.5 cm.

#### Weight

The weight of eligible participants will be taken to help calculate their body mass index (BMI), which is their weight relative to their height, and therefore to determine the prevalence of overweight and obese people in the population. Weight is measured in undergarments using a digital platform scale, to the nearest 100 grams.

#### Waist circumference

Waist circumference is measured to the nearest 0.1 cm using a non-stretchable standard tape measure attached to a spring balance exerting a force of 750 gm. Take measurement over the unclothed abdomen at the smallest diameter between the costal margin and the iliac crest. The tape measure must be kept horizontal for standing measurement. Subject should relax with arms held loosely at sides.

#### Hip circumference

Hip circumference is measured to the nearest 0.1 cm using a non-stretchable standard tape measure attached to a spring balance exerting a force of 750 gm. Take measurement over light clothing at the level of the greater trochanters (usually the widest diameter around the buttocks). The tape measure must be kept horizontal for standing measurement.

### Data analysis

All data forms and questionnaires will be checked for errors and necessary corrections will be made before data entry. Data will be entered using data entry programme with built in range and consistency checks. Frequency distributions will be run to identify outliers. In all analysis, potential confounding variables and effect modifiers will be considered. Descriptive analysis will be performed for all variables and unadjusted comparisons between case and control will be made using T-tests (for continuous variables) or Chi-square Tests (for discrete variables). When appropriate, longitudinal models will be created for outcome variables. All data will be presented before and after adjustment for confounding and interaction. Basic presentations of data will include number and percentages of cases with diabetes by age, sex, location of residence, and in relation to different known risk factors. Chi-square tests will be used to assess the association between diabetes and different risk factors. Prevalence odd ratios will be calculated for diabetes adjusted for major confounding variables (e.g. age, sex, smoking habits, socioeconomic status, nutritional status, and family history of diabetes). Interaction between age and risk factors, obesity and diabetes risk factors, and nutritional status and risk factor (hypertension and diabetes risk factors) will also be examined in the logistic regression model. All statistical analyses will be done using SPSS (Version 20 from SPSS corporation, USA).

Estimation of expenditures for medical care: The mean length of hospital stay and mean payments per admission, per Out Patient Department (OPD) visits, and per purchase of medicine will be calculated. Calculation of annual medical expenditures: Annual rates of use of medical services will be calculated by multiplying the amounts self-reported for the preceding 90 days by 4. We will calculate 90-day OPD expenditures separately for visits made by a participant to hospital clinics, to the private offices of doctors, to pharmacies, and to community health workers.

We will calculate an average daily price, using data about the price paid the most recent time the item was purchased, the number of pills or units of insulin purchased at that time, and the number of pills or units prescribed per day. We will then multiply this result by an adjuster for self-reported adherence, the average number of days per week that the participant indicated that he or she adhered to the prescribed regimen for a given medicine, divided by seven to get the payment per day “as used.” Mean daily payments will be multiplied by 30 to obtain a monthly mean expenditure and by 365 to obtain an annual mean expenditure. Payments for glucose testing strips will be calculated similarly to payments for medicines except that self-reported testing rates (times per day x days per week or month) to be used in lieu of prescribed usage rates and adherence to obtain mean daily, monthly, and annual usage and expenditure.

We will calculate total expenditures for medicalcare per person as the sum of estimated annual payments per person for inpatient hospital admissions, annual payments per person for OPD, annual payments per person for medicines, and annual payments per person for glucose-testing supplies. To avoid double counting, because self-reported payments for OPDs and admissions included payments for medicines, we will subtract from the grand total payments for medicines and strips that were purchased from hospitals during visits and admissions. Based on patient self-report, we will calculate the proportion of hospital, clinic, OPD visits during which medicines were purchased.

Hypothesis Testing: We will use a two-step “hurdle” model to test for differences between persons with DM and persons without DM. Using multivariable logistic regression models that included case status (with DM =1, with Non-DM =0), the team first test for differences in the proportion of subjects with a non-zero value, e.g., persons with any OPD visits during the preceding 90 days, controlling for continuous age, sex, and urban vs. rural residence as well as case. Age will be entered as a linear continuous variable because the addition of other transformations on age, e.g., age squared, will not significantly improve the performance of the model. Then, a second identically specified multivariable model on the non-zero values. The functional form of the second regression model depend on the underlying distribution of values: for counts of admissions, OPDs, and medicines, a Poisson regression model will be used, while for average length of hospital stay and costs per event, we will use ordinary least squares. The hurdle approach does not yield a single overall coefficient or confidence interval for hypothesis testing. However, if the coefficient on DM status is positive and significantly different from zero in one model and positive and not significantly different from zero in the other model, or if both models are significantly positive for DM, then the two models together may be considered statistically significant. As our primary hypothesis is one-sided, we will use one-sided tests to assess significance.

Healthcare use and expenditures: Absolute results for health care use and costs associated with DM can be estimated by subtracting age-, sex-, and location adjusted estimates for persons with Non-DM from the relevant unadjusted results for persons with DM. However, for purposes of comparing results from this study to the results of other studies conducted in other places and times, a more robust statistic is the adjusted ratio of costs or use among persons with DM to those among persons with Non-DM (DM: Non-DM ratio). Ratios are much less influenced than absolute differences by variations in source data, recruitment bias, differences in economic systems, conditions and patterns of medical practice, and currency fluctuations. DM: Non-DM ratios will be calculated. Analysis of use of effective diabetes care: Estimates of the percentage use of essential medicines and other percentage measures of quality and access to medical care will be calculated only for persons with DM. Associations with categories of age and length of time since diagnosis of DM will be tested for statistical significance using multivariable logistic regression models with continuous age, sex, urban vs. rural residence, and continuous duration of DM as predictors.

### Ethics

All participants will be explained about the objectives and importance of the research before recruitment. Participation will be completely voluntary and an appropriate written informed consent will be obtained from all participants. All data-set containing information on the participant will be locked at icddr,b and access to these files will only be given to the PI’s, and the data-manager. Publications and scientific presentations of the findings from the study will be presented in aggregates and identity of individual participants will be kept confidential. The study has received Research Review Committee and Ethical Review Committee clearance from icddr,b PR-13062.

## Discussion

This study will provide an in-depth and comprehensive picture of social and economic impacts of diabetics in Bangladesh and propose clear recommendations for improving prevention and management of diabetes. It will help to develop programs and policies for better management of diabetes and cost effective strategies in Bangladesh context. This study will generate evidence based strategies for formulation policies and programs targeting the general poor patients who cannot afford to pay for the management of diabetes. It will also benefit the women diabetes patients by identifying barriers to access to healthcare, impact of diabetes on individual, family and national levels and its economic impacts and suggest remedies how to overcome these barriers.

The main limitations of this study are the recall bias by cases and controls about several costs associated with disease and hospitalization. Some participants might fail to differentiate costs for treatment of each individual items mentioned in the questionnaire which might affect the results of the study. However, we will perform hospital audits and collect information from multiple sources to verify the correctness of information provided.

The social and economic impact of diabetes study in China shows point-of-service payments or charges are 3.97 times higher among persons with diabetes than among non-diabetes persons, which is higher than published ratios for similar comparisons in developed countries [24]. The study suggests that the human and economic impact of diabetes might be much greater in the developing countries, where 75% of people with diabetes worldwide live, than in the industrialized world where diabetes is diagnosed much earlier and essential effective medicines are more widely prescribed. For a developing country like Bangladesh, the economic costs of diabetes is expected to be huge and with increasing prevalence this will pose a serious threat to the health systems and national economics. As Bangladesh Government has started to decentralize the health systems with inauguration of several primary care centres at the village levels, it is imperative that these structures should be equipped to deal with diabetes screening, primary management and build awareness [25]. Diabetes medication is not available in the health center and health workers are not trained to identify early complications. However, to the best of our knowledge a national action plan for diabetes has not yet been developed by the Government. As diabetes is a costly condition which can be prevented, the results of this study will help allocate resources for prevention and control of diabetes and create greater awareness among policy makers and clinicians.

## Competing interest

All authors declare to not have any conflicts of interest.

## Authors’ contributions

SI, the principal investigator, is involved in concept, design, developing the intervention and the instruments, as well as in the implementation, analysis and reporting aspects of the study. LN was involved in all aspects of the study and provided expert advice for the study design and writing of manuscript. AL, UF and JS are the PhD supervisor for SI and were involved in study design and reviewing the protocol. DSA and GF revised the protocol and provided suggestions to improve the study design and manuscript. All authors have read and approved the final version of the manuscript.

## Authors’ information

SI (MBBS, MPH), is a Senior Research Investigator at the Center for Control of Chronic Diseases, icddr,b and PhD student at the Center for International Health, Ludwig-Maximilians-Universität, Munich, Germany. He specializes in Public Health and Epidemiology with a particular focus on developing solutions for diabetes and other non-communicable diseases.

## Pre-publication history

The pre-publication history for this paper can be accessed here:

http://www.biomedcentral.com/1471-2458/13/1217/prepub

## References

[B1] Zhang P Zhang X Brown J Vistisen D Sicree R Shaw J Nichols G Global healthcare expenditure on diabetes for 2010 and 2030 Diabetes Res Clin Pract 2010 13 293 10.1016/j.diabres.2010.01.026 20171754

[B2] International Diabetes Federation IDF Diabetes Atlas, Fifth Edition 2011 5 Brussels: International Diabetes Federation

[B3] Saleh F Mumu SJ Ara F Begum HA Ali L Knowledge and self-care practices regarding diabetes among newly diagnosed type 2 diabetics in Bangladesh: a cross-sectional study BMC Public Health 2012 13 1112 10.1186/1471-2458-12-1112 23267675 PMC3552981

[B4] Begum S Huda SN Musarrat N Ahmed S Banu LA Ali SM Nutritional status and birth outcomes of the diabetic and non-diabetic pregnant women Bangladesh Med Res Counc Bull 2002 13 3 97 103 14509381

[B5] Ali A Lahiry S Khan AK Ali L Habib SH A downward health care referral system in Bangladesh through diabetic association branches Diabetes Res Clin Pract 2000 13 407 407

[B6] Islam I Mahtab FU Khan AK Ali L Basic care of diabetic patients through self-sustaining approach: a Bangladesh experience Diabetes Res Clin Pract 2000 13 407 407

[B7] Imam T Diabetic prevalence in Bangladesh: the role of some associated demographic and socio-economic characteristics Int J Adv Res Technol, vol 1, no 7, p 95–105 2102 13 95 105

[B8] Jennett PA Hall LA Hailey D Ohinmaa A Anderson C Thomas R Young B Lorenzetti D Scott RE The socio-economic impact of telehealth: a systematic review J Telemed Telecare 2003 13 311 320 10.1258/135763303771005207 14680514

[B9] Javitt JC Chiang Y Economic impact of diabetes Diabetes in America, Bethesda, MD: Natl Inst Health 1995 601 611 http://diabetes.niddk.nih.gov/dm/pubs/america/citation.aspx

[B10] Mohan V Madan Z Jha R Deepa R Pradeepa R Diabetes-social and economic perspectives in the new millenium International journal of diabetes in developing countries 2004 13 29 35

[B11] Zimmet P The burden of type 2 diabetes: are we doing enough? Diabetes Metab 2003 13 6S9 6S18 14502096 10.1016/S1262-3636(03)72783-9 PMC7129589

[B12] Hu FB Globalization of Diabetes The role of diet, lifestyle, and genes Diabetes Care 2011 13 1249 1257 10.2337/dc11-0442 21617109 PMC3114340

[B13] Jonsson B The economic impact of diabetes Diabetes Care 1998 13 C7 C10 9850479 10.2337/diacare.21.3.c7

[B14] Morkrid K Ali L Hussain A Risk factors and prevalence of diabetic peripheral neuropathy: a study of type 2 diabetic outpatients in Bangladesh Int J Diabet Dev Ctries 2010 13 11 10.4103/0973-3930.60004 PMC2859278 20431800

[B15] DAB Statistical Year Book vol. July 2011- June 2012 2012 Dhaka: Diabetic association of Bangladesh

[B16] Bloom DE Canning D The health and wealth of nations Science(Washington) 2000 13 1207 1209 10.1126/science.287.5456.1207 10712155

[B17] Campbell MJ Julious SA Altman DG Estimating sample sizes for binary, ordered categorical, and continuous outcomes in two group comparisons Br Med J 1995 13 1145 10.1136/bmj.311.7013.1145 7580713 PMC2551061

[B18] Coffey JT Brandle M Zhou H Marriott D Burke R Tabaei BP Engelgau MM Kaplan RM Herman WH Valuing health-related quality of life in diabetes Diabetes Care 2002 13 2238 2243 10.2337/diacare.25.12.2238 12453967

[B19] Msyamboza KP Ngwira B Dzowela T Mvula C Kathyola D Harries AD Bowie C The burden of selected chronic non-communicable diseases and their risk factors in Malawi: nationwide STEPS survey PLoS One 2011 13 e20316 10.1371/journal.pone.0020316 21629735 PMC3100352

[B20] Bull FC Maslin TS Armstrong T Global physical activity questionnaire (GPAQ): nine country reliability and validity study J Phys Act Health 2009 13 790 20101923 10.1123/jpah.6.6.790

[B21] Bauman A Craig CL The place of physical activity in the WHO global strategy on diet and physical activity Int J Behav Nutr Phys Act 2005 13 10 10.1186/1479-5868-2-10 16120214 PMC1242353

[B22] Kroenke K Spitzer RL Williams JBW The PHQ-9 J general internal medicine 2001 13 606 613 10.1046/j.1525-1497.2001.016009606.x PMC1495268 11556941

[B23] Martin A Rief W Klaiberg A Braehler E Validity of the brief patient health questionnaire mood scale (PHQ-9) in the general population General hospital psychiatry 2006 13 71 77 10.1016/j.genhosppsych.2005.07.003 16377369

[B24] Yang W Zhao W Xiao J Li R Zhang P Kissimova-Skarbek K Schneider E Jia W Ji L Guo X Medical care and payment for diabetes in China: enormous threat and great opportunity PloS one 2012 13 e39513 10.1371/journal.pone.0039513 23049727 PMC3458850

[B25] Osman FA Health policy, programmes and system in Bangladesh achievements and challenges South Asian Survey 2008 13 263 288 10.1177/097152310801500206

